# Development and Application of a *gp60*-Based Subtyping Tool for *Cryptosporidium bovis*

**DOI:** 10.3390/microorganisms9102067

**Published:** 2021-09-30

**Authors:** Weijian Wang, Muchun Wan, Fang Yang, Na Li, Lihua Xiao, Yaoyu Feng, Yaqiong Guo

**Affiliations:** 1 Center for Emerging and Zoonotic Diseases, College of Veterinary Medicine, South China Agricultural University, Wushan Road, Guangzhou 510642, China; wjwang@stu.scau.edu.cn (W.W.); Muchun_Wan@outlook.com (M.W.); yf1432277601@163.com (F.Y.); nli@scau.edu.cn (N.L.); lxiao1961@gmail.com (L.X.); 2 Guangdong Laboratory for Lingnan Modern Agriculture, Wushan Road, Guangzhou 510642, China

**Keywords:** *Cryptosporidium bovis*, 60-kDa glycoprotein, subtyping tool, genetic diversity, multiple infection episodes

## Abstract

*Cryptosporidium bovis* is a common enteric pathogen in bovine animals. The research on transmission characteristics of the pathogen is hampered by the lack of subtyping tools. In this study, we retrieve the nucleotide sequence of the 60 kDa glycoprotein (GP60) from the whole genome sequences of *C. bovis* we obtained previously and analyze its sequence characteristics. Despite a typical structure of the GP60 protein, the GP60 of *C. bovis* had only 19.3–45.3% sequence identity to those of other *Cryptosporidium* species. On the basis of the gene sequence, a subtype typing tool was developed for *C. bovis* and used in the analysis of 486 *C. bovis* samples from dairy cattle, yaks, beef cattle, and water buffalos from China. Sixty-eight sequence types were identified from 260 subtyped samples, forming six subtype families, namely XXVIa to XXVIf. The mosaic sequence patterns among subtype families and the 121 potential recombination events identified among the sequences both suggest the occurrence of genetic recombination at the locus. No obvious host adaptation and geographic differences in the distribution of subtype families were observed. Most farms with more extensive sampling had more than one subtype family, and the dominant subtype families on a farm appeared to differ between pre- and post-weaned calves, indicating the likely occurrence of multiple episodes of *C. bovis* infections. There was an association between XXVId infection and occurrence of moderate diarrhea in dairy cattle. The subtyping tool developed and the data generated in the study might improve our knowledge of the genetic diversity and transmission of *C. bovis*.

## 1. Introduction

*Cryptosporidium* spp. are important enteric parasites in humans and a variety of animals [1,2,3]. Bovine animals are common hosts of the parasites. In pre-weaned calves, cryptosporidiosis is a major cause of outbreaks of diarrhea [4,5,6,7,8]. In post-weaned calves and adult cattle, subclinical cryptosporidiosis can lead to growth retardation and reduced milk production, respectively [9,10].

*Cryptosporidium bovis* is one of the four common *Cryptosporidium* species in cattle. Previous studies indicated that *C. bovis* mainly infected post-weaned calves in industrialized nations and generally caused asymptomatic infections [11,12]. However, in recent years, *C. bovis* has been commonly found in pre-weaned calves in some Asian and European countries and is responsible for moderate diarrhea [4,5,11,13,14,15]. This difference in the occurrence of *C. bovis* could reflect genetic heterogeneity in infectivity and pathogenicity. Nevertheless, due to the lack of a subtyping tool, little is known of the genetic diversity within the bovine-specific species.

Sequence analysis of the 60 kDa glycoprotein gene (*gp60*) is commonly used in subtyping *Cryptosporidium* spp. [16]. This tool has been used in the identification of infection sources and transmission dynamics of *Cryptosporidium* species in humans and animals [17,18,19,20]. Among the three common intestinal *Cryptosporidium* species in cattle, subtyping tools targeting the *gp60* gene are available for *C. parvum* [21] and *C. ryanae* [22]. Despite the recent sequencing of the genome of *C. bovis* [23], a subtyping tool is yet to be developed for this species.

In this study, we identify the *gp60* gene sequence from the whole genome sequences of *C. bovis* we obtained previously and develop a subtyping tool on the basis of it. The subtyping tool is used in the characterization of *C. bovis* from dairy cattle, beef cattle, yaks, and water buffalos.

## 2. Materials and Methods

### 2.1. Ethics Statement

All fecal samples used in this study were previously collected from cattle with the permission of the farm managers, using procedures in compliance with the Animal Ethics Procedures and Guidelines of the People’s Republic of China. The research protocol was reviewed and approved by the Ethics Committee of the South China Agricultural University.

### 2.2. Samples

DNA preparations from 486 fecal samples of cattle were used in this study, including those from dairy cattle (*n* = 393), yaks (*n* = 73), beef cattle (*n* = 16), and water buffaloes (*n* = 4) (Table 1). These fecal samples were previously collected on 33 farms in nine provinces of China [5,24,25,26,27] and identified as *C. bovis*-positive by PCR and sequence analysis of the *SSU rRNA* gene [28]. The consistency of fecal samples was available from dairy cattle, except those collected from Farm Foshan (*n* = 20). Three categories were assigned to the samples: formed feces with no diarrhea (*n* = 266), loose feces with moderate diarrhea (*n* = 74) and liquid feces with watery diarrhea (*n* = 33). The DNA preparations from the fecal samples were stored at −80 °C until being analyzed by the *C. bovis gp60* PCR.

### 2.3. Identification of the gp60 Sequence of C. bovis

To obtain the nucleotide sequence of the *gp60* gene of *C. bovis*, the whole genome sequences (SRX6096269) of one *C. bovis* isolate ECUST 42482 from a dairy calf in Shanghai, China were downloaded from GenBank [23]. Using the *gp60* sequence (*cgd6_1080*) of *C. parvum* as the reference, the *gp60* gene of *C. bovis* was identified among the whole genome sequences by a blastn analysis. The coding region of the *C. bovis gp60* gene was predicted using FGENESH (http://www.softberry.com/berry.phtml?topic=fgenesh&group=programs&subgroup=gfind, accessed on 9 December 2020) and confirmed by a blastp search of the NCBI database.

### 2.4. PCR of the gp60 Gene

On the basis of the nucleotide sequence of the *gp60* gene, primers were designed to amplify a 1300-bp fragment of the gene using nested PCR. The sequences of primers used were 5′-ATGCGACTTACGCTCTACATTACTCT-3′ (Bovis-*gp60*-F1) and 5′-GACAAAATGAAGG CTGAGATAGATGGGA-3′ (Bovis-*gp60*-R1) in primary PCR, and 5′-CCTCTCGG CATTTATTGCCCT-3′ (Bovis-*gp60*-F2) and 5′-ATACCTAAGGCCAAATGCTGATGAA-3′ (Bovis-*gp60*-R2) in secondary PCR. The PCR reaction was 50 µL in volume, which contained 1 µL DNA (for primary PCR) or 2 µL primary PCR product (for secondary PCR), 0.25 µM primary primers or 0.5 µM secondary primers and 1 × PCR master mix (Thermo Scientific, Waltham, MA, USA). In addition, 400 ng/µL nonacetylated bovine serum albumin (Sigma-Aldrich, St. Louis, MO, USA) was used in primary PCR to reduce PCR inhibitors. The amplification program consisted of a pre-denaturation at 94 °C for 5 min, 35 cycles of 94 °C for 45 s, 55 °C for 45 s, and 72 °C for 90 s, and a final extension at 72 °C for 10 min. The secondary PCR products were visualized under UV light after 1.5% agarose gel electrophoresis.

### 2.5. Sequence Analysis

Positive secondary PCR products were sequenced bidirectionally on an ABI 3730 Genetic Analyzer at Sangon Biotech Co. (Shanghai, China). The raw sequences from each PCR product were assembled using the software Chromosol 1.5 (Technelysium Pty Ltd, Brisbane, Australia), and edited using the software BioEdit 7.05 (Bioedit Ltd, Manchester, UK). The sequences obtained were aligned with the reference sequence from the whole genome assembly using the software ClustalX 2.1 (Development supported by Science Foundation Ireland, http://www.clustal.org/, accessed on 6 May 2021). They were used to construct a maximum likelihood tree to assess their genetic relatedness using the software MEGA 7.0 (Development supported by National Institutes of Health (United States), https://www.megasoftware.net/, accessed on 6 May 2021) and substitution rate was calculated on the basis of the general time-reversible model. The reliability of cluster formation was evaluated using the bootstrap method with 1000 replicates. The sequences in each well-supported cluster were considered as belonging to the same subtype family. Nucleotide sequence identity among subtype families was calculated using BioEdit 7.05 (Bioedit Ltd, Manchester, UK), and potential recombination events were identified on the basis of sequences of segregating sites (excluding insertions and deletions) using the software DnaSP 5.10 (Universitat de Barcelona, Barcelona, Spain). The nucleotide sequences were further translated into amino acid (AA) sequences and aligned with GP60 sequences of *C. hominis*, *C. parvum*, *C. viatorum*, *C. ubiquitum*, *C. ryanae*, and *C. xiaoi* using ClustalX 2.1. The signal peptide and glycosylphosphatidylinositol (GPI) anchor in GP60 sequence were predicted using PSORT II (http://psort.hgc.jp/form2.html, accessed on 11 December 2020). N- and O- glycosylated sites were predicted using NetNGlyc 1.0 (http://www.cbs.dtu.dk/services/NetNGlyc/, accessed on 11 December 2020) and YinOYang 1.2 (http://www.cbs.dtu.dk/services/YinOYang/, accessed on 11 December 2020) servers, respectively. The furin cleavage site was predicted using ProP 1.0 (http://www.cbs.dtu.dk/services/ProP/, accessed on 11 December 2020).

### 2.6. Nucleotide Sequence Accession Numbers

The complete *gp60* gene of *C. bovis* and representative nucleotide sequences of 68 sequence types of the *C. bovis* *gp60* gene obtained in this study were deposited in GenBank under the accession numbers MZ977132-MZ977200.

### 2.7. Statistical Analysis

The Chi-square test implemented in the software IBM SPSS Statistics 25 (International Business Machines Corp., Armonk, NY, USA) was used to compare differences in *C. bovis* subtype family occurrence between dairy cattle with or without diarrhea. Differences were considered significant when *p* < 0.05.

## 3. Results

### 3.1. Characteristics of the gp60 Gene of C. bovis

**Figure 1:**
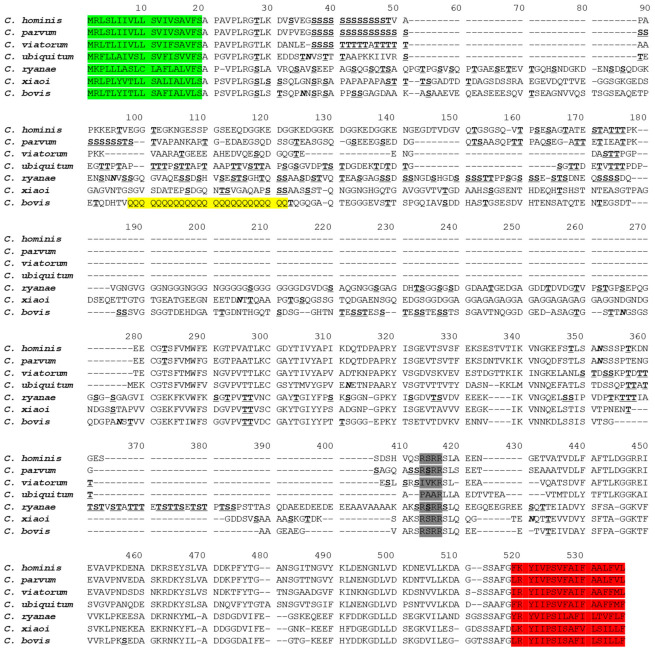
Alignment of the full GP60 sequence from *Cryptosporidium bovis* (this study) with those from *C. hominis* (ACQ82748), *C. parvum* (AAF78281), *C. viatorum* (AJP62575), *C. ubiquitum* (XP_028874367), *C. ryanae* [23], and *C. xiaoi* (QXJ78680). The signal peptide at the N-terminus and GPI anchor at the C-terminus are shaded in green and red, respectively. The putative furin cleavage site is shaded in gray. Potential N-linked glycosylation sites are marked in bold and italics, and O-linked glycosylation sites in bold with underline. The unique polyglutamine tract in *C. bovis* is highlighted in yellow. Dashes indicate amino acid deletions.

The complete *gp60* gene (GenBank No. MZ977200) was identified in contig_176 of the whole genome sequences of *C. bovis*. The coding region of the gene consisted of 1353 nucleotides encoding 450 amino acids. The GP60 of *C. bovis* had only 19.3–45.3% sequence identity to those of *C. hominis* (ACQ82748), *C. parvum* (AAF78281), *C. viatorum* (AJP62575), *C. ubiquitum* (XP_028874367), *C. ryanae* [23], and *C. xiaoi* (QXJ78680) (Figure 1). However, the GP60 of *C. bovis* possessed a signal peptide at the N-terminus and a glycosylphosphatidylinositol (GPI) anchor at the C-terminus, which are common in GP60 proteins of other *Cryptosporidium* species. A conserved amino acid sequence RSRR for the furin cleavage site was present between the GP40 and GP15 fragments of the protein. In the GP40 fragment of *C. bovis*, there was a polyglutamine tract encoded by CAA/CAG trinucleotide repeats, instead of the polyserine tract encoded by TCA/TCG/TCT trinucleotide repeats in *C. parvum* and *C. hominis*. Altogether, three potential N-glycosylation sites and 36 O-glycosylation sites were identified in the GP60 of *C. bovis* (Figure 1).

### 3.2. Amplification Efficiency of the Subtyping Tool

Altogether, 294 (60.5%) of the 486 DNA preparations from *C. bovis* generated the expected *gp60* PCR products, included 262 from dairy cattle, 27 from yaks, 4 from beef cattle, and one from water buffalo (Table 1). The amplification efficiency of the PCR using DNA preparations were stored for less than three years (samples from Heilongjiang, Hebei, Jiangsu, Yunnan, and Guangdong) (77.7% or 181/233) was much higher than that using DNA preparations that were stored for a longer period (samples from Qinghai, Henan, Shanghai, and Hunan) (44.7% or 113/253) (Table 1). Among the latter, 20 were randomly selected for re-analysis of the DNA using the *SSU rRNA*-based nested PCR [28], with only one being confirmed as still positive for *C. bovis*.

### 3.3. Sequence Polymorphism in the C. bovis gp60 Gene

Of the 294 *gp60* PCR products, 260 were sequenced successfully using the secondary primers, which generated 68 sequence types, including one type identical to the reference from whole genome sequencing. Overall, nucleotide sequence differences of 0.1–49.3% were present among the sequence types. The sequence types differed from each other by both single nucleotide polymorphism (SNP) and length polymorphism (indel). The maximal difference in length among the sequence types was 138 bp. The SNPs and indel were scattered across the *gp60* gene.

### 3.4. Nomenclature and Phylogenetic Relationship of C. bovis Subtype Families

**Figure 2:**
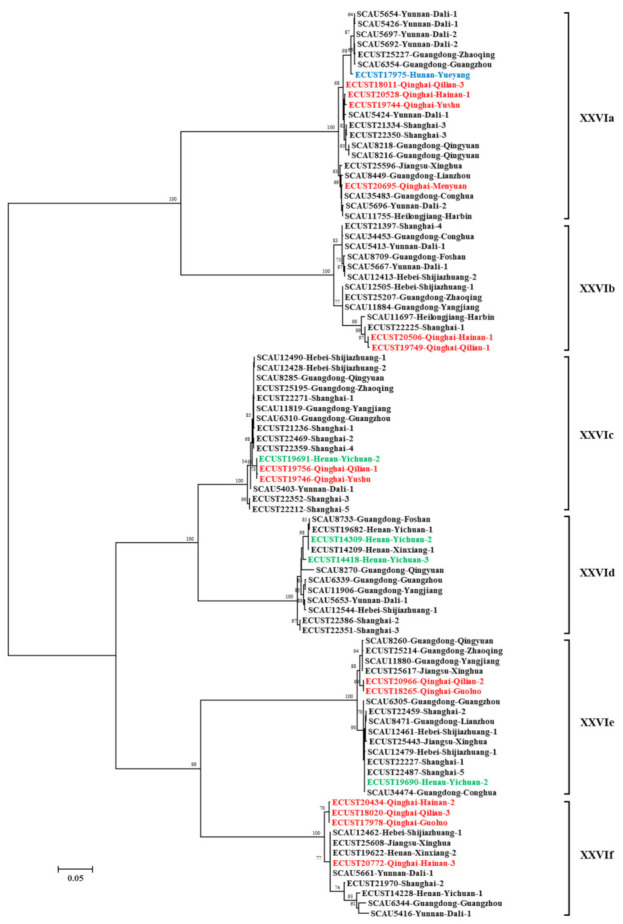
Phylogenetic relationship among six *Cryptosporidium bovis* subtype families (XXIVa–XXIVf) inferred by a maximum likelihood analysis of partial sequences of the *gp60* gene. Sequences from dairy cattle, beef, yaks, and water buffalo are indicated by black, green, red, and blue characters, respectively. Bootstrap values greater than 50% from 1000 replicates are shown on branches. The scale bar equals 5 nucleotide substitutions per 100 nucleotides.

**Figure 3:**
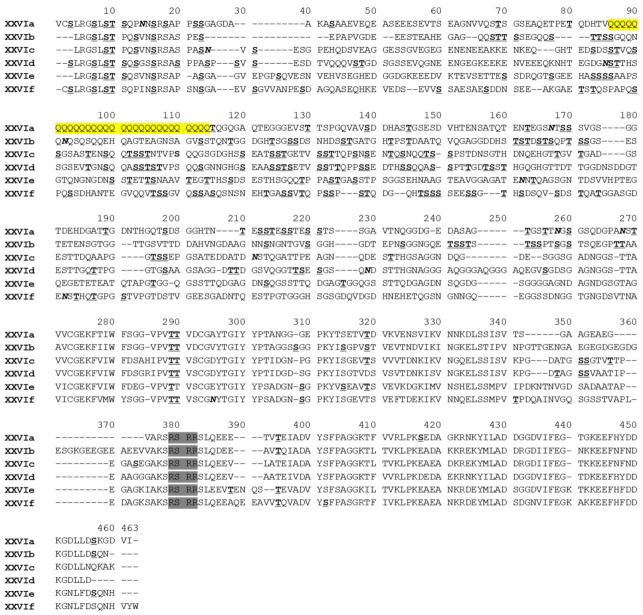
Alignment of partial amino acid sequences of GP60 from six *Cryptosporidium bovis* subtype families. The putative furin cleavage site is shaded in gray. Potential N-linked glycosylation sites are marked in bold and italics, and O-linked glycosylation sites in bold with underline. The unique polyglutamine tract in XXIVa is highlighted in yellow. Dashes indicate amino acid deletions, except those at the N- and C-termini of the sequences.

On each farm, one representative of each *gp60* sequence type was used in phylogenetic analysis. The 90 sequences selected formed six reliable clusters in a maximum likelihood tree. According to the nomenclature of *gp60* subtype families, each cluster was designated as a subtype family and named as XXVIa, XXVIb, XXVIc, XXVId, XXVIe, and XXVIf (Figure 2). Among them, XXVIa and XXVIb formed a group divergent from the other group formed by the other four subtype families. In agreement with this, the nucleotide identity between XXVIa/XXVIb and other subtype families was low (50.7–59.9%) (Table 2). The nucleotide differences among the subtype families included SNPs and indels across the entire *gp60* sequences. However, sequences at the 5′ and 3′ ends were more conserved among the subtype families. For examples, XXVIb had a high sequence identity to XXVIe and XXVIc at the 5′ and 3′ end of the sequence, respectively. XXVIe was very similar to XXVIf at the 3′ end of the sequence. This suggested the occurrence of genetic recombination among subtype families. In a DnaSP analysis, 121 potential recombination events were identified among all the six subtype families. At the amino acid level, sequence polymorphism among six of the subtype families was most obvious in the N-terminus of the GP40 fragment (Figure 3). In contrast, sequences were more conserved at the C-terminus of GP40 and the entire GP15 region. Except for XXVIa, the other subtype families did not have a polyglutamine tract encoded by CAA/CAG in GP40.

### 3.5. Distribution of Subtype Families by Host and Age

The six subtype families were all identified in dairy cattle in the order of XXVIc (55), XXVIe (50), XXVIb (45), XXVIa (44), XXVId (23), and XXVIf (12). In yaks, five of the six subtype families were detected, with XXVId absent (Table 1). The dominant subtype family in yaks was XXVIf (9/26), which was less common (12/229) in dairy cattle. In beef cattle, three subtype families were obtained from the four successfully subtyped samples, including XXVId (2), XXVIc (1), and XXVIe (1). The single sample from the water buffalo subtyped belonged to XXVIa.

Overall, the six subtype families were found in pre-weaned as well as in post-weaned dairy calves. However, on a particular farm, the distribution of subtype families in pre-weaned dairy calves appeared different from that in post-weaned dairy calves (Table 3). On Farm Dali-1, pre-weaned dairy calves were infected by XXVIb (6), XXVIf (4), XXVId (2), and XXVIa (1). However, in post-weaned dairy calves on the farm, XXVIf and XXVId disappeared, while XXVIc (4) emerged. Subtype family alteration was also observed on Farms Shijiazhuang-1, Xinghua, Guangzhou, and Yangjiang (Table 3). On some other farms, including Shanghai-1, Shanghai-4, and Foshan, the subtype families switched completely between pre-weaned and post-weaned dairy calves (Table 3).

### 3.6. Distribution of C. bovis Subtype Families by Farm and Location

One to five subtype families were identified on each of the 33 study farms, with nine farms having one subtype family, 12 farms having two, five farms having three, four farms having four, and three farms having five (Table 1). On the farms with one subtype family, only one to three samples were successfully subtyped, with the exception of Farm Dali-2 where nine samples were subtypes. In contrast, farms with multiple subtype families mostly had reasonable numbers of samples subtyped. Among the farms with one or two subtype families, some had the same distribution of subtype families, such as Farms Dali-2, Menyuan and Yueyang, which all had only XXVIa. On the farms with three or more subtype families, the distribution of *C. bovis* subtype families varied by farm (Table 1).

In each province, one to six subtype families were identified (Table 4). In Shanghai and Guangdong, all six subtype families were observed. However, the subtype family distribution in these two provinces was different, with XXVIc (41/63) being dominant in Shanghai and XXVIe (42/97) being dominant in Guangdong. A similar situation was observed in the provinces with five subtype families (Hebei, Qinghai, and Yunnan). One to four subtype families were present in Hunan, Jiangsu, Heilongjiang, and Henan.

### 3.7. Corrlation between C. bovis Subtype Families and Diarrhea Occurance in Dairy Cattle

Of the 373 fecal samples with data on fecal consistency, 214 were subtyped, including those from cattle with no diarrhea (*n* = 149), moderate diarrhea (*n* = 43), and watery diarrhea (*n* = 22) (Table 5). All six subtype families were observed in the three groups of dairy cattle. However, the frequency of XXIVd in dairy cattle with moderated diarrhea (18.6%, 8/43) was significantly higher than that in dairy cattle with no diarrhea (6.7%, 10/149; χ^2^ = 0.018, *p* = 0.018). In contrast, for the other five subtype families, the frequency of the subtype family in cattle with no diarrhea was not significantly different form that in dairy cattle with moderated diarrhea and watery diarrhea, respectively (Table 5).

## 4. Discussion

In this study, we identified the *gp60* gene from a published genome of *C. bovis* and established a *gp60*-based subtyping tool. Using the tool, a highly genetic diversity was revealed in *C. bovis* from dairy cattle, beef cattle, yaks, and water buffalos. The data obtained suggest that there could be potential differences in *C. bovis* subtypes between pre-weaned and post-weaned dairy cattle.

Results of comparative analysis indicate that *C. bovis* is significantly different from other *Cryptosporidium* species at the *gp60* gene. The length of *gp60* gene in *C. bovis* was ~1353 bp, which is much longer than those in *C. parvum*, *C. hominis*, *C. meleagridis*, and other species (873–1089 bp) [21,29,30,31,32,33], but similar to those in the genetically related *C. xiaoi* (~1437 bp) and *C. ryanae* (~1548 bp) [18,22]. Although the GP60 of *C. bovis* is structurally similar to those of other species, its sequences for the N-terminal signal peptide and C-terminal GPI anchor are unique. This makes it difficult to use the *gp60* primers for other species, which are generally based on sequences in the two regions, to amplify the *gp60* gene of *C. bovis* by PCR. In addition, a large number of SNPs are present in the *gp40* fragment between *C. bovis* and other *Cryptosporidium* species (Figure 1). Since GP40 targets host cell receptors during sporozoite invasion, the sequence differences in GP40 fragment could contribute to biological differences between *C. bovis* and other species [21].

The *gp60* PCR assay developed here could be used in studies of the transmission of *C. bovis*. In the study, 294 of 486 *C. bovis*-positive DNA preparations produced expected amplicons using the *gp60* PCR we developed. The overall amplification efficiency of the PCR is 60.5%. The true amplification efficiency could be much higher, as some DNA preparations used might have degraded during long storage. This was supported by the greatly reduced amplification efficiency of the PCR (44.7%) when DNA preparations stored for more than three years were used in PCR. The 20 randomly selected *gp60* PCR-negative DNA preparations were mostly negative for *Cryptosporidium* in the re-analysis of the DNA using an *SSU rRNA*-based PCR. Importantly, a high genetic diversity was observed within *C. bovis* in sequence analysis of the *gp60* PCR products, producing 68 sequence types from 260 samples. Therefore, the *gp60* subtyping tool has high resolution, thus could be effective in genetic characterization of *C. bovis*.

The high genetic diversity within *C. bovis* is likely due to the wide occurrence of genetic recombination. In addition to the numerous SNPs among the six subtype families identified, mosaic sequences were observed, especially among XXVIb, XXVIc, and XXVIe. The 121 potential recombination events in the obtained *gp60* sequences further supported the occurrence of frequent genetic recombination at the locus in *C. bovis*. The high prevalence and long duration of *C. bovis* infection and frequent occurrence of multiple subtype families on a single farm could greatly facilitate the occurrence of genetic recombination among isolates, leading to the emergence of new subtypes. This was in agreement with the observations in *C. xiaoi* and *C. ryanae* [18,22]. Several studies on other *Cryptosporidium* species also indicated the frequent genetic recombination at the *gp60* locus [34,35].

No clear host adaption was observed within *C. bovis*. All six subtype families of *C. bovis* have been observed in dairy cattle. These subtype families have been mostly identified in other bovine animals. In yaks, only five subtype families were observed, but the number of samples examined was small and the absent subtype family XXVId was detected in beef cattle. In addition, the other two subtype families detected in beef cattle (XXVIc and XXVIe) are common subtype families in dairy cattle. The only subtype family in water buffalo, XXVIa, is also common in dairy cattle and yaks. Thus, no obvious differences were observed in the distribution of *C. bovis gp60* subtype by host. This was different from the observations in other *Cryptosporidium* species, including the genetically related *C. xiaoi* and *C. ryanae* [18,22,36].

Differences in the transmission dynamics of *C. bovis* among geographic locations are not obvious currently. Among the three provinces with significant numbers of *C. bovis* samples (Yunnan, Shanghai, and Guangdong), the dominant subtype families identified were mostly similar (Table 4). In Yunnan, the dominant one among the five identified subtype families was XXVIa, which was identified at reasonable frequency in Guangdong and Shanghai. Although the most commonly identified subtype family in Guangdong, XXVIe, was not detected in dairy cattle in Yunnan, it has been detected in Shanghai and other areas. The lack of geographic segregation of subtype families is expected in view of the high prevalence of *C. bovis* and frequent genetic recombination at this locus.

There could be age-associated differences in the distribution of *C. bovis* subtype families on farms. On some farms, the dominant subtype family in pre-weaned calves disappeared or became less common in post-weaned calves. Accordingly, there were usually some new subtype families appearing in the post-weaned calves on these farms. As GP60 is an immunodominant antigen involved in the invasion of *Cryptosporidium* spp., the changes in *gp60* subtype families between young and older calves could reflect the existence of subtype-specific immunity after an initial episode of *C. bovis* infection. Further studies involving longitudinal sampling of animals and subtyping of isolates from sequential episodes are needed to resolve this important issue.

*Cryptosporidium bovis* subtype families may have different degrees of associations with diarrhea. As indicated by results of the χ^2^ analysis, five of the six *C. bovis* subtype families were not significantly associated with moderate or watery diarrhea in dairy cattle. This was expected, as *C. bovis* infection had no obvious association with diarrhea in dairy cattle in most previous studies [13,25,37]. However, in the present study, the frequency of XXVId was significantly higher in dairy cattle with moderate diarrhea. This supports the observation in a few studies that *C. bovis* has modest pathogenicity in dairy cattle [38,39].

## 5. Conclusions

We identified the *gp60* sequence of *C. bovis* and developed a subtyping tool for the pathogen on the basis of the sequence acquired. Using the tool, we showed a high genetic diversity within *C. bovis*, and a lack of host-associated differences in the distribution of the six subtype families were identified. The distribution of subtype families on a farm could be different between pre- and post-weaned calves, due to possible subtype-specific immunity. Future studies using more samples from different regions and hosts and sequence analyses of additional genetic loci are needed to further characterize the genetic diversity and transmission dynamics of *C. bovis*.

## Figures and Tables

**Table 1:** Sources and subtype family identity of *Cryptosporidium bovis* samples used in the study.

Host	Province	Farm	No. of Samples Analyzed	No. of Samples Positive by *gp60* PCR (%)	No. of Samples Subtyped	Subtype Family (No. of Samples)
Dairy cattle	Heilongjiang	Harbin	10	10 (100.0)	9	XXVIa (8), XXVIb (1)
	Hebei	Shijiazhuang-1	14	11 (78.6)	10	XXVIb (3), XXVId (3), XXVIe (2), XXVIc (1), XXVIf (1)
		Shijiazhuang-2	6	5 (83.3)	4	XXVIb (3), XXVIc (1)
	Henan ^1^	Yichuan-1	14	2 (14.3)	2	XXVId (1), XXVIf (1)
		Xinxiang-1	5	1 (20.0)	1	XXVId (1)
		Xinxiang-2	4	1 (25.0)	1	XXVIf (1)
	Jiangsu	Xinghua	13	10 (76.9)	10	XXVIa (6), XXVIe (3), XXVIf (1)
	Shanghai ^1^	Shanghai-1	61	38 (62.3)	37	XXVIc (35), XXVIb (1), XXVIe (1)
		Shanghai-2	30	17 (56.7)	8	XXVId (5), XXVIc (1), XXVIe (1), XXVIf (1)
		Shanghai-3	18	8 (44.4)	7	XXVIa (4), XXVIc (2), XXVId (1)
		Shanghai-4	16	8 (50.0)	8	XXVIb (7), XXVIc (1)
		Shanghai-5	12	6 (50.0)	3	XXVIc (2), XXVIe (1)
	Yunnan	Dali-1	40	30 (75.0)	23	XXVIb (9), XXVIa (4), XXVIc (4), XXVIf (4), XXVId (2)
		Dali-2	10	9 (90.0)	9	XXVIa (9)
	Guangdong	Zhaoqing	28	18 (64.3)	17	XXVIe (12), XXVIc (3), XXVIa (1), XXVIb (1)
		Guangzhou	25	21 (84.0)	17	XXVIe (10), XXVIf (3), XXVId (2), XXVIa (1), XXVIc (1)
		Yangjiang	20	17 (85.0)	17	XXVIe (8), XXVIb (5), XXVIc (3), XXVId (1)
		Foshan	20	15 (75.0)	15	XXVIb (14), XXVId (1)
		Qingyuan	18	13 (72.2)	12	XXVId (6), XXVIa (3), XXVIe (2), XXVIc (1)
		Conghua	15	12 (80.0)	11	XXVIe (8), XXVIa (2), XXVIb (1)
		Lianzhou	14	10 (71.4)	8	XXVIa (6), XXVIe (2)
	subtotal		393	262 (66.7)	229	XXVIc (55), XXVIe (50), XXVIb (45), XXVIa (44), XXVId (23), XXVIf (12)
Yak	Qinghai ^1^	Guoluo	10	6 (60.0)	6	XXVIf (4), XXVIe (2)
		Qilian-1	13	4 (30.8)	4	XXVIb (2), XXVIc (2)
		Qilian-2	12	2 (16.7)	1	XXVIe (1)
		Qilian-3	9	2 (22.2)	2	XXVIa (1), XXVIf (1)
		Menyuan	10	3 (30.0)	3	XXVIa (3)
		Hainan-1	6	2 (33.3)	2	XXVIa (1), XXVIb (1)
		Hainan-2	4	1 (25.0)	1	XXVIf (1)
		Hainan-3	3	3 (100.0)	3	XXVIf (3)
		Yushu	6	4 (66.7)	4	XXVIc (3), XXVIa (1)
	subtotal		73	27 (37.0)	26	XXVIf (9), XXVIa (6), XXVIc (5), XXVIb (3), XXVIe (3)
Beef cattle	Henan ^1^	Yichuan-2	11	3 (27.3)	3	XXVIc (1), XXVId (1), XXVIe (1)
		Yichuan-3	5	1 (20.0)	1	XXVId (1)
	subtotal		16	4 (25.0)	4	XXVId (2), XXVIc (1), XXVIe (1)
Water buffalo	Hunan ^1^	Yueyang	4	1 (25.0)	1	XXVIa (1)
Total			486	294 (60.5)	260 (53.5)	XXVIc (61), XXVIe (54), XXVIa (51), XXVIb (48), XXVId (25), XXVIf (21)

^1^ The DNA preparations of these samples were stored for more than three years before being used in the study.

**Table 2:** Percent nucleotide sequence identity among *Cryptosporidium bovis* subtype families at the *gp60* locus.

	XXVIa	XXVIb	XXVIc	XXVId	XXVIe	XXVIf
XXVIa	-					
XXVIb	54.9–59.6	-				
XXVIc	51.4–53.9	56.5–59.9	-			
XXVId	53.7–56.7	52.1–56.3	75.3–77.7	-		
XXVIe	51.2–54.3	52.1–57.1	59.2–64.4	58.1–62.3	-	
XXVIf	50.7–54.0	51.9–55.1	62.1–65.8	58.0–62.6	62.8–65.8	-

**Table 3:** Distribution of *Cryptosporidium bovis* subtype families at the gp60 locus in pre- and post-weaned dairy calves on study farms.

Farm	Pre-Weaned Dairy Calves		Post-Weaned Dairy Calves	
	No. of Samples Subtyped	Subtype Family (No. of Samples)	No. of Samples Analyzed	Subtype Family (No. of Samples)
Harbin	9	XXVIa (8), XXVIb (1)	-	
Shijiazhuang-1	1	XXVId (1)	6	XXVIb (3), XXVId (2), XXVIc (1)
Shijiazhuang-2	4	XXVIb (3), XXVIc (1)	-	
Yichuan-1	-		1	XXVId (1)
Xinxiang-1	1	XXVId (1)	-	
Xinxiang-2	1	XXVIf (1)	-	
Xinghua	1	XXVIe (1)	5	XXVIa (3), XXVIe (2)
Shanghai-1	35	XXVIc (35)	2	XXVIb (1), XXVIe (1)
Shanghai-2	8	XXVId (5), XXVIc (1), XXVIf (1), XXVIe (1)	-	
Shanghai-3	7	XXVIa (4), XXVIc (2), XXVId (1)	-	
Shanghai-4	7	XXVIb (7)	1	XXVIc (1)
Shanghai-5	3	XXVIc (2), XXVIe (1)	-	
Dali-1	13	XXVIb (6), XXVIf (4), XXVId (2), XXVIa (1)	10	XXVIc (4), XXVIa (3), XXVIb (3)
Dali-2	9	XXVIa (9)	-	
Zhaoqing	17	XXVIe (12), XXVIc (3), XXVIa (1), XXVIb (1)	-	
Guangzhou	9	XXVIe (8), XXVIc (1)	8	XXVIf (3), XXVId (2), XXVIe (2), XXVIa (1)
Yangjiang	12	XXVIe (6), XXVIb (3), XXVIc (3)	5	XXVIb (2), XXVIe (2), XXVId (1)
Foshan	14	XXVIb (14)	1	XXVId (1)
Qingyuan	-		12	XXVId (6), XXVIa (3), XXVIe (2), XXVIc (1)
Conghua	11	XXVIe (8), XXVIa (2), XXVIb (1)	-	
Lianzhou	8	XXVIa (6), XXVIe (2)	-	
Total	170	XXVIc (48), XXVIe (39), XXVIb (36), XXVIa (31), XXVId (10), XXVIf (6)	51	XXVId (13), XXVIa (10), XXVIe (9), XXVIb (9), XXVIc (7), XXVIf (3)

**Table 4:** Distribution of *Cryptosporidium bovis* subtype families at the *gp60* locus by location in China.

Host	Province	No. of Samples Subtyped	Subtype Family (No. of Samples)
Dairy cattle	Heilongjiang	9	XXVIa (8), XXVIb (1)
	Hebei	14	XXVIb (6), XXVId (3), XXVIe (2), XXVIc (2), XXVIf (1)
	Henan	4	XXVId (2), XXVIf (2)
	Jiangsu	10	XXVIa (6), XXVIe (3), XXVIf (1)
	Shanghai	63	XXVIc (41), XXVIb (8), XXVId (6), XXVIa (4), XXVIe (3), XXVIf (1)
	Yunnan	32	XXVIa (13), XXVIb (9), XXVIc (4), XXVIf (4), XXVId (2)
	Guangdong	97	XXVIe (42), XXVIb (21), XXVIa (13), XXVId (10), XXVIc (8), XXVIf (3)
Yak	Qinghai	26	XXVIf (9), XXVIa (6), XXVIc (5), XXVIb (3), XXVIe (3)
Beef cattle	Henan	4	XXVId (2), XXVIc (1), XXVIe (1)
Water buffalo	Hunan	1	XXVIa (1)
Total	-	260	XXVIc (61), XXVIe (54), XXVIa (51), XXVIb (48), XXVId (25), XXVIf (21)

**Table 5:** Difference in the frequency of *Cryptosporidium bovis* subtype families in dairy cattle with different diarrhea statues.

Subtype Family	No. of Samples Subtyped	No. of Samples without Diarrhea	Moderate Diarrhea				Watery Diarrhea		
			*n*	χ ^2^	OR (95% CI) ^1^	*p*	χ ^2^	OR (95% CI)	*p*
XXVIa	44	35	6	1.807	0.528 (0.206–1.355)	0.179	1.077	0.514 (0.144–1.841)	0.299
XXVIb	31	22	8	0.373	1.319 (0.541–3.218)	0.541	1.720	0.275 (0.035–2.149)	0.190
XXVIc	55	33	15	2.887	1.883 (0.901–3.934)	0.089	1.000	1.640 (0.618–4.357)	0.317
XXVId	22	10	8	5.556	3.177 (1.168–8.644)	0.018 ^2^	3.355	3.089 (0.877–10.881)	0.067
XXVIe	50	41	5	4.624	0.347 (0.128–0.942)	0.032	0.861	0.585 (0.187–1.833)	0.353
XXVIf	12	8	1	0.692	0.420 (0.051–3.452)	0.406	2.177	2.783 (0.679–11.407)	0.140
Total	214	149	43	-	-	-	-	-	-

^1^ OR: odds ratio; CI: confidence interval. ^2^ Significant *p* value < 0.05.

## Data Availability

The representative nucleotide sequences obtained in this study are openly available in GenBank under the accession numbers MZ977132-MZ977200.
